# Effect of high slice energy spread of an electron beam on the generation of isolated, terawatt, attosecond X-ray free-electron laser pulse

**DOI:** 10.1038/s41598-020-57905-y

**Published:** 2020-01-28

**Authors:** Chi Hyun Shim, Yong Woon Parc, Dong Eon Kim

**Affiliations:** 1 Pohang Accelerator Laboratory, Pohang University of Science and Technology, Pohang, 37673 Korea; 2 Division of Advanced Nuclear Engineering, Pohang University of Science and Technology, Pohang, 37673 Korea; 3 Department of Physics, Center for Attosecond Science and Technology, Pohang University of Science and Technology, Pohang, 37673 Korea; 4 Max Planck POSTECH/KOREA Res. Init., Pohang, 37673 Korea

**Keywords:** Free-electron lasers, X-rays, X-rays

## Abstract

Attosecond (asec) X-ray free-electron laser (XFEL) has attracted considerable interest over the past years. Nowadays typical XFEL application experiments demand 10^10^–10^11^ photons per pulse, which corresponds to a peak power of terawatts (TW) in case of asec hard X-ray pulse. To the realization of such TW asec-XFEL pulse, however, the unavoidable increase of slice energy spread (SES) due to laser heater, which is commonly used to mitigate the micro-bunching instability (MBI), would be a major obstacle. To deal with this problem, the effect of such a SES is investigated in this work. The results reveal that (1) SES of a current spike is linearly proportional to the peak current of a current spike in an electron beam, (2) surprisingly, this linearity is independent of the wavelength of an energy modulation driving laser which is used to make a current spike and (3) the gain length of current spike in the undulator is sensitive to the initial SES, so there is an optimal peak current of the current spike for successful FEL lasing process. Utilizing these characteristics, a series of simulations with parameters for Pohang Accelerator Laboratory X-ray Free Electron Laser was carried out to demonstrate that an isolated, TW asec-XFEL pulse can be generated even when the SES is increased due to the usage of laser heater to prevent the MBI in the XFEL. We show that an isolated X-ray pulse with >1 TW and a pulse duration of 73 as (~3 × 10^10^ photons/pulse at 12.4 keV or 0.1 nm) can be generated by using ten current spikes with optimal peak current. It becomes clear for the first time that the disadvantage from the increased SES can be indeed overcome.

## Introduction

Direct measurement and control of electron dynamics in their time scale in atomic, molecular and nanoscopic systems have been cherished since the dawn of quantum mechanics in the 20^th^ century. The last two decades have observed that such cherished dreams are coming closer to the realization due to the advance of attosecond (asec) metrologies based on medium-scale^1–6^ and large-scale lasers^7,8^ and the advent of X-ray free electron laser (XFEL) sources^9–13^. These new novel sources are opening up new frontiers in ultrafast extreme ultraviolet (XUV) and X-ray science. This new paradigm will be even more enriched with the development of isolated asec pulses with higher power.

Advanced ultrashort sources based on optical lasers cover the visible to XUV (a few to ~100 eV) with a pulse duration of a few femtoseconds down to a few tens of asec. High-order harmonic generation (HHG) sources can generate a photon pulse in the XUV region (10~40 nm) with a pulse duration of hundreds of asec or less^14,15^. Such XUV sources have been used in the study of electron dynamics in atoms and molecules such as imaging and control of a two-electron wave packet, time-resolved dynamics of Fano resonance and Auger process, and continue to be demanded^15^. However, we note that it is hard to expect high-intensity X-ray pulses from laser-based asec sources due to the cut-off photon energy and the low conversion efficiency of HHG process. The tunability of photon energy in laser-based asec sources is also rather limited; however, the accessibility to these sources is easier and more available than that to XFEL.

**Figure 1:**
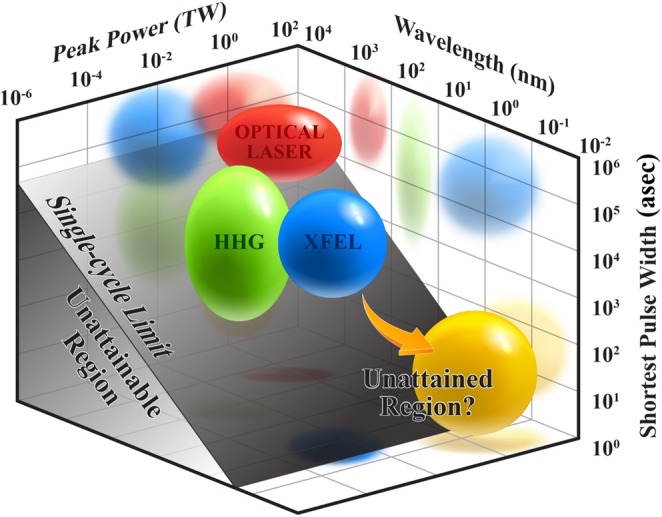
Current ultrafast photon sources in the view of pulse duration, wavelength and pulse power. HHG: high-order harmonic generation; XFEL: X-ray free-electron laser.

XFEL-based sources can generate high-intensity asec X-ray pulse due to the large extraction efficiency from the electron beam. However, such a source has not been achieved until now as shown in Fig. 1. The development of such high-intensity asec X-ray sources will greatly extend the realm of ultrafast processes that can be explored and may enable single-molecule imaging^16^. The realization of single-molecule imaging is a holy grail of imaging science. Furthermore, intense isolated asec-XFEL pulses can observe real-time changes in the probability distribution of an electron’s position^17,18^, and could clarify the boundary between quantum mechanical and classical contributions. This technology could also enable four-dimensional imaging with picometer spatial resolution and asec temporal resolution^17^. Current intense XFEL pulses and peta-watt lasers can routinely produce bizarre states of matter such as inner shell holes in atoms^19^ or even hollow atoms, and warm dense matter^20^. Investigation on the dynamics for these new states of matter requires an intense isolated asec-XFEL, and will open up a field of dynamical X-ray nonlinear science^21^. While the accessibility to XFEL is very limited, XFEL provides continuous tunability of photon energy and sufficient number of photons per pulse, which is more suitable to the investigation of nonlinear phenomena in ultrafast time scale in X-ray region. We can conclude that laser-based asec source and XFELs have complementary nature for investigating the ultrafast phenomena. While laser-based asec sources will be suitable to investigations involving ultrafast XUV spectroscopy, XFEL based asec sources will be better fit to the studies utilizing ultrafast X-ray spectroscopy. These new, noble sources will work together to enhance and deepen our understanding of nature in the areas that have never been explored before.

Experimental and theoretical studies have been devoted to how to generate asec X-ray pulses from XFELs. Experimental approaches include X-ray Laser-Enhanced Attosecond Pulses (XLEAP) by the Linac Coherent Light Source (LCLS)^22,23^, which has succeeded in producing asec-XFEL in the soft X-ray region^24^. European X-ray Free Electron Laser and Pohang Accelerator Laboratory (PAL) have also shown interest in asec-XFEL projects^25,26^. Ref. ^27^ provides a good review of the proposals^28–33^ for TW asec X-ray pulse: multiple current spikes with equally-spaced distance^28^, an irregularly spaced slotted foil^29^, and the tilt of electron beam for shorter X-ray pulses^30^. If we make irregularly spaced current spikes in the electron beam, the slotted foil and the X-ray delay unit are not necessary for the generation of an isolated TW asec X-ray pulse^31^. A novel idea of re-using a single current spike by using small X-ray delay units between the undulators was tested out^32^. It was later demonstrated in simulation that the isolated TW asec X-ray pulse can be obtained by using a single current spike even without the X-ray delay units^33^. This proposal is the simplest one among the proposed methods up to now for the TW-level asec X-ray pulse generation.

Only recently it was pointed out that large slice energy spread (SES, see ‘Methods’ section for the definition of SES) of an electron beam due to the usage of a laser heater to mitigate the micro-bunching instability (MBI) may be a major obstacle to obtaining an asec X-ray pulse with power >1 TW^27^. Although there are several other causes for the increase of SES such as nonlinear energy chirp in an electron beam, cathode condition of the electron gun and etc., laser heater is most dominant cause of the increase of SES^34^ and relevant to the topic of the current spike investigation. The MBI originates from the space charge effect in the electron beam and the coherent radiation at a chicane type bunch compressor^35^. All of the XFELs suffer from the MBI, and they use laser heaters to mitigate MBI, which inevitably introduces high energy spread to the electron beam^36–39^. A laser heater can reduce MBI during operation of XFEL^40^ at the cost of an increase in the SES of the electron beam (Supplementary Information in more detail); this increase is expected to result in a current spike even with a larger SES in enhanced self-amplified spontaneous emission (E-SASE) method^41^. This large SES will impose difficulties on the amplification of radiation power to TW-level. Nevertheless, the effect of increased SES has not been seriously considered in the previous studies of the generation of a TW asec-XFEL pulse. The following important questions related to the SES should be addressed: (1) how the SES depends on the wavelength *λ*_L_ of the modulation laser used to make current spike, (2) how seriously the increase of SES limits the peak value of a current spike, (3) how badly the increase limits the amplification of radiation power or affects the gain length of current spike in the undulator and (4) the possibility about TW-power asec X-ray pulse generation in the presence of large SES.

In this paper, we address all the questions and demonstrate by simulation that a method for TW asec-XFEL pulse can be devised even under large SES, showing the generation of a ~73 asec, isolated XFEL pulse at 12.4 keV with >1 TW peak radiation power. This demonstration has been possible after the systematic investigation of the relationship between the SES and the peak current *I*_*P*_ of current spike, and by the relationship between the gain length and *I*_*P*_ of current spike. This is the first study that includes properly the effect of large SES in the generation of an isolated, TW, asec-XFEL pulse. Though the parameters of Pohang Accelerator Laboratory X-ray Free Electron Laser (PAL-XFEL) are used in this paper, the results are applicable to other XFEL facilities.

## Simulation Results

### Slice energy spread issues

In this section, we present simulation results that quantify the dependence of SES of a current spike on wavelength of a modulation laser (*λ*_L_) used in E-SASE method, on the peak value of a current spike (*I*_*P*_), and on the SES of the initial electron beam; we also demonstrate how SES affects the gain length of a current spike for amplification.

**Figure 2:**
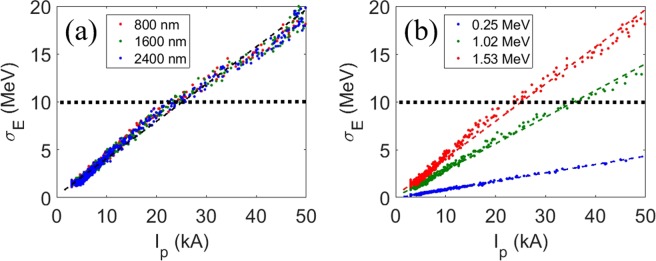
RMS SES (σ_*E*_) of the current spike vs. peak current of the spike for (**a**) different laser wavelengths *λ*_*L*_ at a base SES of 1.53 MeV and (**b**) different base SESs at *λ*_*L*_ = 800 nm and a base current of 3 kA. Blue dots are for a base SES of 0.25 MeV (ref. ^33^) with a fitting of σ_*E*_ = 0.0879*I*_P_ − 0.0620 by regression (blue dashed line). Green dots are for a base SES of 1.02 MeV with a fitting of σ_*E*_ = 0.2783*I*_P_ + 0.0591 regression (green dashed line). Red dots are for a SES of 1.53 MeV (as in Fig. 2(a)). Horizontal black dashed lines represent the limit of SES of the current spike, below which an appropriate lasing process is expected from Eq. (1) (the ‘Methods’ section for the detail simulation process).

To increase the peak current of the current spike while preserving the width of current spike, the electrons must be bunched from a region as wide as possible in the electron beam (see ‘Methods’ section). For this reason, a long wavelength (*λ*_L_) of a modulation laser is preferred. Hence a series of simulations with different *λ*_L_ has been done and their results are plotted in Fig. 2(a) (red dots for *λ*_L_ = 0.8 μm, green for *λ*_L_ = 1.6 μm, blue for *λ*_L_ = 2.4 μm). The SES of the base electron beam was fixed at 1.53 MeV, which is the nominal value for PAL-XFEL when the laser heater is under operation. We note that the SES (σ_*E*_) increases linearly with *I*_*P*_: σ_*E*_ = 0.3890*I*_*p*_ + 0.1979, and has no dependency on λ_L_.

The slope of the SES curve with *I*_*P*_ (*λ*_L_ = 800 nm) increases as the SES of a base electron beam increases (Fig. 2(b)). The base current of the electron beam is set at 3 kA, which is a nominal value in PAL-XFEL. The fitting result for the red dots is the same as that in Fig. 2(a). Figure 2(b) reveals that if the SES of a base electron beam is high, the SES of the current spike is also high.

Horizontal black dashed lines indicate the limit of SES of the current spike. The limit is defined by using the Pierce parameter *ρ* which is calculated as^42^:

1 $$\rho ={\left[\left(\frac{{I}_{{\rm{p}}}}{{I}_{{\rm{A}}}}\right){\left(\frac{{\lambda }_{{\rm{w}}}{A}_{{\rm{w}}}}{2\pi {\sigma }_{{\rm{x}}}}\right)}^{2}{\left(\frac{1}{2{\gamma }_{0}}\right)}^{3}\right]}^{1/3},$$

where *I*_A_ = 17.045 kA the Alfven current, *λ*_w_ is the undulator period, *A*_w_ the undulator parameter, σ_x_ the beam size and *γ*_0_ the Lorentz factor. To obtain a sufficient gain in the undulator, the SES of a current spike should satisfy the criteria^43^: $\frac{{\sigma }_{{\rm{E}}}}{{\rm{E}}} < \rho$, where the FEL parameter *ρ* ≈ 10^−3^. The electron beam energy in PAL-XFEL is 10 GeV; therefore, σ_E_ must be <10 MeV (horizontal black dashed lines, Fig. 2).

At a glance, FEL lasing efficiency could be increased by increasing *I*_P_ , however, the increase of *I*_P_ also increases SES, and this change in turn decreases the gain. As a result, counter-intuitively, the lasing can be degraded^44,45^. Thus, there must be an optimum value for the peak current (*I*_P_) of the current spike. To find an optimal the peak current (*I*_P_) of current spike related to the gain length of current spike, Ming Xie’s fitting formula^46,47^ is utilized.

The one-dimensional gain length^44,45^ is calculated from *ρ* as:

2 $${L}_{1{\rm{d}}}={\lambda }_{{\rm{w}}}/4\pi \sqrt{3}\rho .$$

Then the three-dimensional (3D) gain length (*L*_g_) normalized by the one-dimensional gain length can be expressed by a scaling function as^46–49^:

3 $$\frac{{L}_{{\rm{g}}}}{{L}_{1{\rm{d}}}}=1+\eta ({\eta }_{{\rm{d}}},{\eta }_{\epsilon },{\eta }_{{\rm{\gamma }}}),$$

where *η* is a scaling function^46^ that is the function of a diffraction parameter ${\eta }_{{\rm{d}}}=\frac{{L}_{1{\rm{d}}}}{{L}_{{\rm{r}}}}$, an emittance parameter ${\eta }_{\epsilon }=(\frac{{L}_{1{\rm{d}}}}{\beta })(\frac{4\pi {\epsilon }}{\lambda })$, and an energy-spread parameter ${\eta }_{{\rm{\gamma }}}=4\pi (\frac{{L}_{1{\rm{d}}}}{{\lambda }_{{\rm{w}}}})(\frac{{\sigma }_{{\rm{E}}}}{{E}_{0}})$. The Rayleigh length *L*_r_ in the diffraction parameter is defined as ${L}_{{\rm{r}}}=4{\rm{\pi }}{\sigma }_{{\rm{x}}}^{2}/\lambda$, where *λ* is the wavelength of the X-ray pulse.

**Figure 3:**
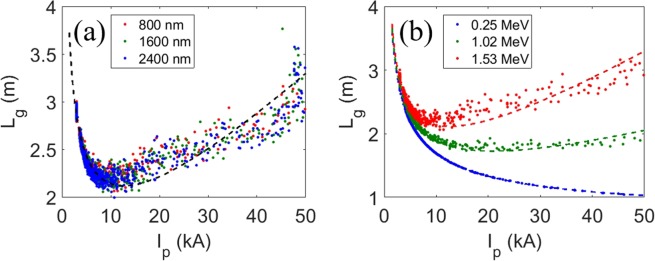
The 3D gain length with respect to the peak current for (**a**) different modulation laser wavelengths for a base SES of 1.53 MeV, and (**b**) different SESs of the base electron beam. Dashed lines: Eq. (3) with linear fitting result from Fig. 2 for the rms SES σ_E_.

By using Eq. (3), the 3-D gain lengths for the cases in Fig. 2(a) were calculated and shown in Fig. 3(a). *L*_*g*_ initially decreases for the increase of *I*_P_ as intuitively expected. However, for *I*_P_ > ~ 10 kA, counter-intuitively *L*_g_ increases again because the SES becomes so high that the total gain in X-ray output power is less than at *I*_P_ = 10 kA. As expected, *L*_g_ does not depend on *λ*_L_. Hence the important parameter to decrease the gain length is *I*_P_ itself, not *λ*_L_.

The dependence of *L*_g_ on *I*_P_ was calculated (Fig. 3(b)) for the cases in Fig. 2(b). When the base SES is sufficiently low (0.25 MeV), a high *I*_P_ is preferable to reduce *L*_g_ (Fig. 3(b), blue dots). However, as the base SES increases, the smallest *L*_g_ occurs at an optimal *I*_P_ (Fig. 3(b), green and red dots). Hence we decided to use *I*_P_ = 10 kA for this simulation, because the SES of the base current in PAL-XFEL is ~1.5 MeV when the laser heater is under operation.

This investigation has revealed that SES increases linearly as the peak current (*I*_P_)of the current spike increases, but is independent of *λ*_L_. The slope of the increase of SES with respect to *I*_P_ is sensitive to the SES of the base current. The gain length has an optimum value when the SES of the base current is high. Hence while SES should be minimized during the generation, acceleration and manipulation stages of the electron beam, the optimal peak current of current spike should be identified for a given SES for the best amplification of asec-XFEL.

### Generation of isolated terawatt attosecond X-ray pulse

#### Single current spike case

**Figure 4:**
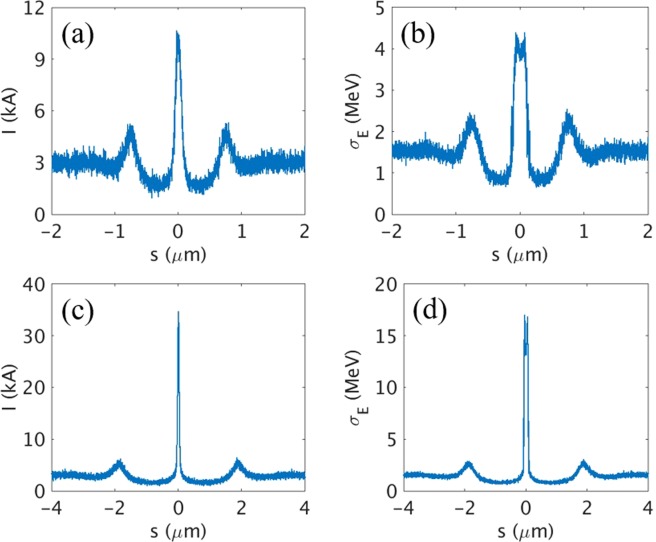
(**a**) Current profile and (**b**) SES profile of the electron beam for the case of single current spike with 10 kA peak current. (**c**) Current profile and (**d**) SES profile of the electron beam for the case of single current spike with 35 kA peak current.

In this section, we discuss whether the optimal peak current of current spike has to be used and whether an isolated asec-XFEL with TW peak-power can be generated even under the high SES with a single current spike^33^. The two representative single current spikes with optimal peak current (10 kA, Fig. 4(a),(b)) and quite high peak current (35 kA, Fig. 4(c),(d)) were generated by using the E-SASE method. For the case of a 1.5-cycle modulation laser at *λ*_L_ = 800 nm and ΔE = 10.2 MeV, the peak current (*I*_P_) of the main current spike reaches up to 10 kA with a current spike width of 120 nm FWHM (Fig. 4(a)). The profile of SES (σ_E_) along the electron beam for all the slices are plotted in Fig. 4(b). Note that the SES at the main *I*_P_ position is lower than 5 MeV as shown in Fig. 2. To increase *I*_P_ of the current spike while maintaining its spike width, *λ*_L_ should be increased with ΔE being increased simultaneously^33^, and therefore *λ*_L_ = 2000 nm and ΔE = 51.1 MeV is used. In this case, *I*_P_ of the current spike is increased up to 35 kA (Fig. 4(c)). As the peak current of current spike is increased, the SES is also increased to about 15 MeV (Fig. 4(d)) which is also recognized in Fig. 2.

**Figure 5:**
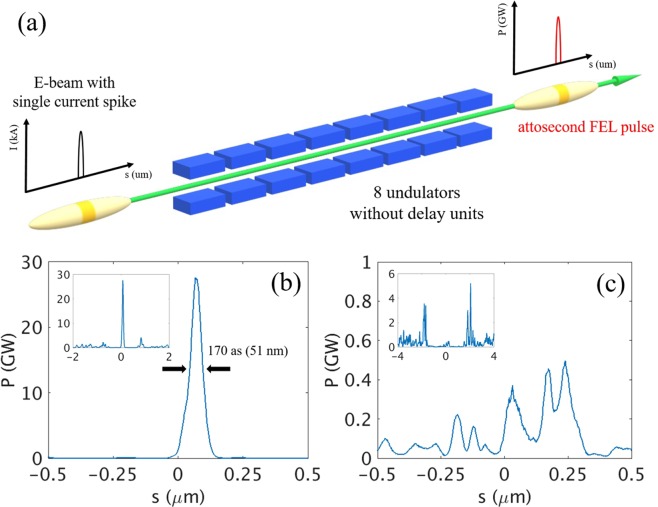
(**a**) Undulator layout for the radiation amplification by a single current spike. (**b**) Expanded temporal profile of radiation at the end of 8^th^ undulator for the main current spike in Fig. 4(a) (10 kA case). (**c**) Expanded temporal profile of radiation at the end of 8^th^ undulator for the main current spike in Fig. 4(c) (35 kA case). Inset in (**b,c**): temporal profile of total XFEL pulse.

With the two representative cases in Fig. 4, the simulations for radiation were performed by using GENESIS 1.3 code^50^. Layout of undulator line for the case of single current spike is shown in Fig. 5(a). From the single current spike of 10 kA, the radiation power of up to 28 GW at 12.4 keV with a pulse duration of 170 asec FWHM (Fig. 5(b)) is generated for PAL-XFEL undulator parameters (Table S1 in Supplementary Information). Although the generated XFEL pulse had side peaks (Fig. 5(b), inset), their intensities are rather smaller than main radiation pulse. From the single current spike of 35 kA, on the other hand, a power of only 0.4 GW is generated at the position of the current spike (Fig. 5(c)) and the background electrons radiate more than those in the current spike (Fig. 5(c), inset).

At the optimal value for the peak current of the current spike, the gain length of the current spike is minimized and the power growth of the radiation from the current spike is faster than that from the other portion of the electron beam. Therefore, the XFEL pulse radiated from the current spike with optimal peak current can be an isolated pulse as shown in Fig. 5(b). However, when the high peak current is used for current spike neglecting the negative effect of SES, a spiky long XFEL pulse is typically generated as shown in Fig. 5(c). The radiation power radiated from other parts of the electron beam is even higher than that from the current spike because the gain length of other parts can be shorter than that of current spike due to large SES of current spike as shown in Fig. 3. The simulation results reveal that in spite of the increased SES, a single current spike with optimal peak current can generate 170 asec isolated XFEL pulse to a level of 30 GW power but not to a level of >1 TW radiation power.

#### Multi current spikes case

**Figure 6:**
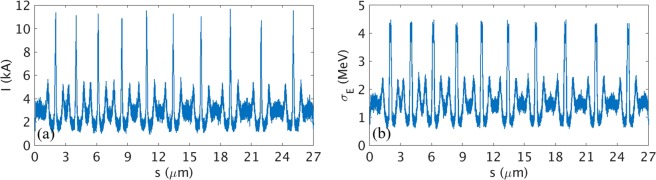
(**a**) Current profile and (**b**) SES profile of the electron beam for the case of multi current spikes with 10 kA peak current.

To overcome the significant increase of SES and generate TW-level asec-XFEL pulse, the multi current spike method^31^ is examined in this subsection. To generate multi current spikes in the electron beam from the E-SASE method, we use a multi-cycle laser pulse with an energy chirp^31^. Because of the chirp in the modulation laser, the multi current spikes are separated by increasing distances (Fig. 6(a)) which is suitable for generating isolated intense pulse^31^. The total charge of the electron beam including the base level current is 270 pC. The total length of the electron beam is 27 μm and the number of current spikes is ten. Peak current of each spikes is about 10 kA and the current spikes have SES <5 MeV (Fig. 6(b)).

**Figure 7:**
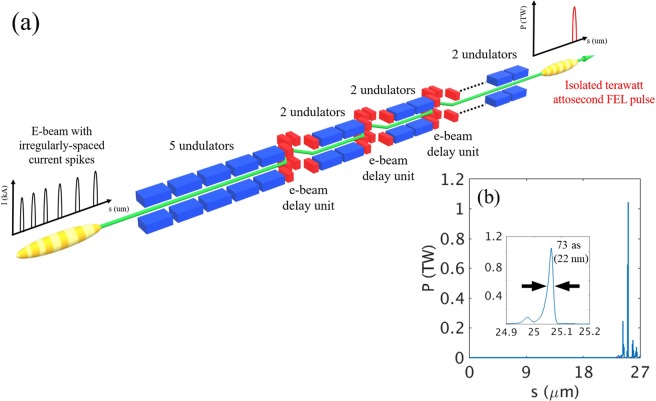
(**a**) Undulator section layout for multi current spike method. Red blocks: electron beam delay units between undulators. (**b**) Temporal profile of XFEL pulse with 23 undulators; inset: expanded view of main radiation pulse.

The simulation considered ten current spikes as shown in Fig. 6(a), with a total of 23 undulators: five in the first stage and two for each of the nine stages. To obtain a sufficient initial radiation power, five undulators are assigned in the beginning of the undulator line as one unit before the first electron beam delay unit (Fig. 7(a), red blocks). In the later sections, two undulators as one unit are sufficient to maintain the fast amplification of radiation power between the electron beam delay units. For different XFEL machines, the number of undulators between the delay units can be adjusted. The delay units can match a target X-ray pulse (generated from one current spike) to the new current spike just ahead of the X-ray pulse. This is repeated in each delay unit. Then the target X-ray pulse can always see a new fresh current spike in each undulator^31^.

With this configuration, an isolated TW asec-XFEL pulse can be produced (Fig. 7(b)). The pulse width is ~73 asec (FWHM) and the peak power is >1 TW (Fig. 7(b), inset). We used only 23 undulators in this study due to the space limitation of PAL-XFEL undulator hall; for other XFEL facilities, the number of undulators can be changed. We conclude that the multi current spikes method^31^ can produce an isolated, TW-level asec-XFEL pulse even under the malignant effect by the increased SES.

## Summary and Discussion

Large SES is fatal to the realization of a TW asec-XFEL pulse. Due to MBI, a laser heater at an injector part must be used during normal XFEL operation, but the laser heater increases the initial SES of the electron beam, and this SES impedes the generation of a TW asec-XFEL pulse. To devise a way to produce an isolated, TW asec-XFEL, the characteristics of the current spike has been explored. The key observations are: (1) SES increases linearly as the peak current (*I*_P_) of a current spike increases but not on *λ*_L_, and (2) the rate of increase is sensitively dependent on the initial amount of SES. (3) The gain length (*L*_g_) of amplification process in the undulator is also affected by SES, so there exists an optimal *I*_P_ for a current spike in the FEL lasing process; this optimal *I*_P_ is ~10 kA for PAL-XFEL. Note that 10 kA is specific value for PAL-XFEL; however, there would be another optimum value for each XFEL facility.

Two series of simulations were performed: one for a single current spike case, and the other for a multi current spikes case. In the case of a single current spike (10 kA), XFEL pulse with only 28GW radiation power and 170 asec pulse duration (~2.5 ×10^9^ photons/shot at 12.4 keV or 0.1 nm) is generated by using 8 undulator units. To produce the XFEL pulse of up to 1 TW with a pulse duration of 73 asec (~3 ×10^10^ photons/shot at 12.4 keV or 0.1 nm), ten current spikes in the electron beam with optimal peak current is necessary for PAL-XFEL. We demonstrate and devise a way by simulation that an isolated TW asec-XFEL pulse can be generated even when the SES is large due to a laser heater.

## Methods

**Figure 8:**
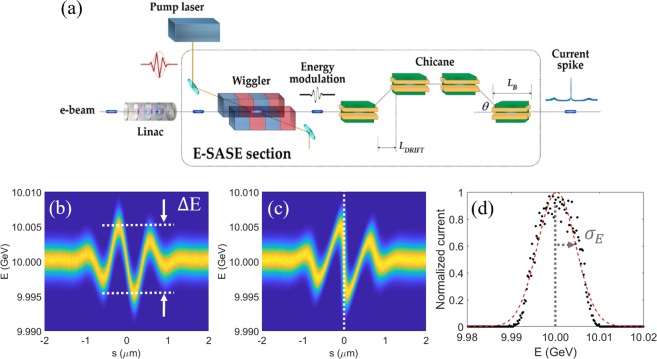
(**a**) Schematic view of the E-SASE method. Energy distribution of an electron beam (**b**) after wiggler and (**c**) after chicane in the E-SASE section. (**d**) Energy distribution (black dots) of the electrons in a selected slice depicted by a white vertical dashed line in Fig. 8(c); red line: fitting line with Gaussian distribution.

This section outlines the enhanced self-amplified spontaneous emission (E-SASE) method (Fig. 8(a))^41^ to make current spike in an electron beam. A few-cycle laser is sent to the wiggler synchronously with an electron beam, and the interaction between the laser and the electron beam under the magnetic field of a wiggler yields a particle distribution with energy modulation (ΔE, Fig. 8(b)). This energy modulation gets steeper and is converted to a density modulation by a subsequent chicane (Fig. 8(c)). This density modulation represents, in general, current spikes. The particle distribution is divided by equally spaced slices along the propagation direction of the electron beam. The current profile along the electron beam can be obtained by integrating the particle distribution in each slice (i.e., by integrating Fig. 8(c) over the energy axis). The energy distribution of a sliced section (white dashed line in Fig. 8(c)) is plotted in Fig. 8(d) and the rms SES (σ_E_) of the slice is defined as the rms value of the distribution function (red dashed line in Fig. 8(d)). By controlling the parameters related to the modulation laser and the electron beam, appropriate current shape can be obtained for specific purpose; a single current spike or multi current spikes, which is essential for the generation of an isolated TW asec-XFEL pulse.

## Supplementary information

Supplementary Information .
