# Supplementary material for: Effect of high slice energy spread of an electron beam on the generation of isolated, terawatt, attosecond X-ray free-electron laser pulse
Source: Sci Rep. 2020 Jan 28;10:1312. doi: 10.1038/s41598-020-57905-y (PMC6987125; doi:10.1038/s41598-020-57905-y)
Supplement: Supplementary file 1 — Supplementary Information . [file 41598_2020_57905_MOESM1_ESM.pdf]

Supplementary Information for

Effect of high slice energy spread of an electron beam on the generation of isolated, terawatt, attosecond X-ray free-electron laser pulse

Chi Hyun Shim[1], Yong Woon Parc[1,2*], and Dong Eon Kim[3,4#]

[1]*Pohang Accelerator Laboratory, Pohang University of Science and Technology, Pohang 37673, Korea*

[2]*Division of Advanced Nuclear Engineering, Pohang University of Science and Technology, Pohang 37673, Korea*

[3]*Department of Physics, Center for Attosecond Science and Technology, Pohang University of Science and Technology, Pohang 37673, Korea*

[4]*Max Planck POSTECH/KOREA Res. Init., Pohang 37673, Korea*

E-mail: (*) young1@postech.ac.kr, (#) kimd@postech.ac.kr

Micro-bunching instability (MBI)

An electron beam has an inherent density fluctuation along the propagation direction as a result of the variations in the photo-electron emission process by a laser pulse at the photo-cathode of an electron gun. This density fluctuation is caused by fluctuation in the spatial and temporal characteristics of a driving laser (a UV laser, typically), and by the roughness and inhomogeneity in a photocathode surface. The density fluctuation is randomly distributed and results in different space charge forces between the electrons; as a consequence, energy varies along the electron beam. This energy modulation induces additional density modulation when the electron beam passes through a chicane-type bunch compressor. An electron will feel two effective forces, one from electrons in front of itself, the other from those behind of itself. The difference in the electron densities results in the difference in the effective forces. Thus, the electron will gain or lose energy; this process induces a further change in energy modulation. This energy modulation causes another density modulation in the next bunch compressor in the linac; this process is repeated in the linac of the XFEL. This growing process of the density modulation is called 'micro-bunching instability (MBI)', and is fatal to XFEL operation [35].

The ELEGANT [S1] simulation clearly shows the MBI in an electron beam at the end of linac for PAL-XFEL case (Fig. S1a). In PAL-XFEL, a strong coherent radiation in the 3rd bunching compressor has been observed, using a visible charge-coupled-device (CCD) camera in a diagnostic section after the 3rd bunching compressor [S2]. The change in the coherent radiation strength was observed when the power of a laser heater was controlled.

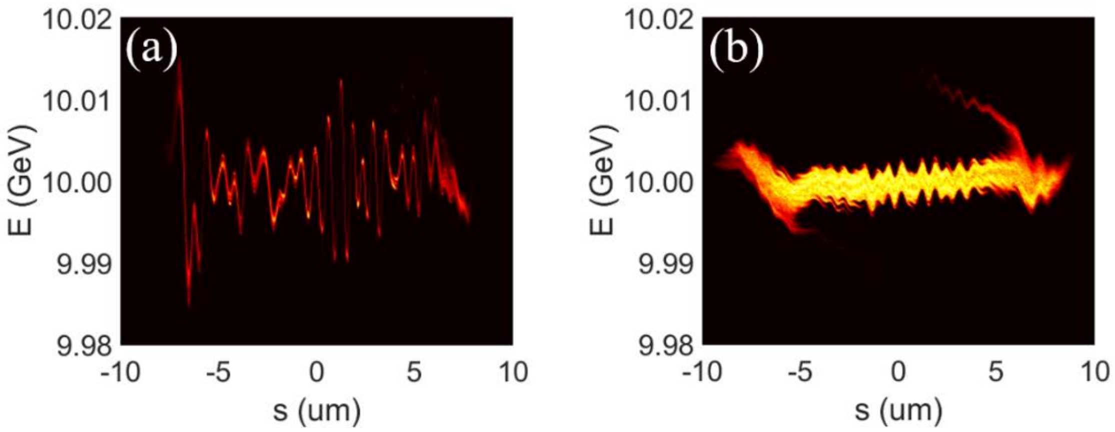

Fig. S1: Energy distribution of the electron beam at the end of linac in the PAL-XFEL for the two cases of (a) laser heater OFF and (b) laser heater ON at a peak laser power of 30 kW.

The distribution (Fig. S1(a)) is too noisy to permit the generation of a single current spike by a chicane-type bunch compressor. A laser heater [40] has been typically used to clean up the distribution (Fig. S1(b)). We note that the irregularity of the distribution is considerably reduced at the cost of increased slice energy spread (SES). If the laser power is too high, the SES of the electron beam increases too much, so the current spike has a very large SES. PAL-XFEL has SES of up to 1.5 MeV. Such a high SES will prevent the efficient lasing that is required for the generation of a terawatt-level attosecond X-ray pulse.

[S1] Borland, M. Elegant: A flexible SDDS-Compliant code for accelerator simulation. *Advanced Photon Source* LS-287 (2000).

[S2] Ko, J. H., Kim, G., Kim, C., Kang, H.-S., & Ko, I. S. Coherent synchrotron radiation monitor for microbunching instability in XFEL. *Rev. Sci. Instrum*. **89**, 063302 (2018).

PAL-XFEL parameters

The parameters used for the generation of the current spike with the E-SASE method at the end of linac for PAL-XFEL are listed in Table S1.

Table S1. Main parameters used in simulations.

Parameter		Value	Unit
Electron beam at the end of linac			
Energy		10	GeV
Charge	(Normal / single-current)	200	pC
	(Multi-current method)	270	pC
Base current		3	kA
rms normalized slice emittance		0.4	μm
rms slice energy spread		1.5	MeV
Laser heater section			
Undulator Period		50	mm
Number of periods		9	
Laser wavelength		760	nm
Laser peak power		30	kW
E-SASE section			
Wiggler period		0.544	m
Number of periods of wiggler		2	
amplitude of the bunch compressor of the chicane		207.58 (10 kA)<br>129.65 (35 kA)<br>264.60 (multi-current)	μm
Laser wavelength		800 (10 kA)<br>2000 (35 kA)<br>800 (multi current)	nm
Number of cycles of laser pulse		1.5	
Energy modulation		10.2 (10 kA)<br>51.1 (35 kA)<br>10.2 (multi current)	MeV
Undulator section			
Undulator period		26	mm
Wavelength (Photon energy)		0.1 (12.4)	nm (keV)
